# Season of birth/C-reactive protein gene interaction differentially affects negative symptoms domains in patients with schizophrenia

**DOI:** 10.1192/j.eurpsy.2025.1529

**Published:** 2025-08-26

**Authors:** V. Golimbet, T. Lezheiko, M. Gabaeva, N. Kolesina, V. Mikhailova

**Affiliations:** 1 Clinical Genetics Laboratory, Mental Health Research Center, Moscow, Russian Federation

## Abstract

**Introduction:**

Schizophrenia is a severe psychiatric disease caused by genetic and environmental factors or their interactions that can contribute across multiple disease domains, including negative symptoms (NS), a core feature of schizophrenia.

**Objectives:**

To study the association between season of birth (SOB), a well-replicated risk factor for schizophrenia, and NS domains avolition/apathy (AA) and diminished expression (DE) and to search for an interaction effect of SOB and rs2794521 genetic variants of the inflammatory marker C-reactive protein (CRP) on these domains.

**Methods:**

The study included 2475 patients with schizophrenia. Patients born during the months of December to February were considered to be winter-born (n=636) and patients born in other months were considered to be non-winter-born (n=1839). Genotypes for CRP rs2794521 were obtained for 2437 patients. NS factors were calculated based on the Positive and Negative Syndromes Scale.

**Results:**

There was a significant effect of SOB on AA scores (p=0.009), which remained after adjustment for sex and illness duration. Patients born in winter had higher scores compared with those born in other seasons. No significant effect of SOB on DE scores was observed. An association between the CRP rs2794521 *G*-allele and AA scores was found (p=0.044) in the winter-born group, with the carriers of the *G*-allele having higher scores. There was no effect of the *G* allele on DE scores in this group and on AA or DE scores in the non-winter group.

**Conclusions:**

The results provide new evidence about the effect of SOB and SOB/CRP gene interaction on schizophrenia NS domains.

**Disclosure of Interest:**

None Declared

